# Gentle Touch and Sucrose for Pain Relief during Suctioning in Preterm Newborns—A Randomized Clinical Trial

**DOI:** 10.3390/children10010158

**Published:** 2023-01-13

**Authors:** Nayara Rodrigues Gomes de Oliveira, Cibelle Kayenne Martins Roberto Formiga, Bruna Abreu Ramos, Rafaela Noleto dos Santos, Nayara Nubia de Sousa Moreira, Patricia Gonçalves Evangelista Marçal, Waldemar Naves do Amaral

**Affiliations:** 1 Graduate Program in Health Sciences, Medical School, Federal University of Goiás (UFG), Goiânia 74690-900, GO, Brazil; 2 Program in Sciences Applied to Health Products, Department of Physical Therapy, State University of Goiás (UEG), Goiânia 74690-900, GO, Brazil

**Keywords:** pain, preterm, interventions, gentle touch, sucrose

## Abstract

Pain management is challenging in neonatal care. We aimed to compare the effects of gentle touch and sucrose on pain relief during suctioning in premature newborns (PTNB). This crossover randomized clinical trial enrolled PTNBs with low birth weight, hemodynamically stable, and requiring suctioning during hospitalization in the neonatal intensive care unit. PTNBs underwent three different suctioning procedures. The first was performed without intervention (baseline). Right after, PTNBs were randomly allocated (sucrose and gentle touch or vice versa) to the next two suctioning procedures. Two validated scales assessed pain: the Neonatal Infant Pain Scale (NIPS) and the Premature Infant Pain Profile-Revised (PIPP-R). We evaluated 50 PTNBs (mean of 28 weeks) with a mean low birth weight of 1050 g; most were under continuous positive airway pressure 37 (74%) and mechanical ventilation 41 (82%). Gentle touch was efficacious for pain relief since NIPS (*p* = 0.010) compared to baseline. Sucrose was also effective in reducing pain NIPS and PIPP-R (*p* < 0.001). Although the two interventions reduced pain, no difference was observed between gentle touch and sucrose.

## 1. Introduction

Before the 1980s, it was believed that newborns did not feel pain. However, this concept contrasts with studies showing that newborns feel pain and are hypersensitive to painful stimuli due to nervous system immaturity [1]. Although the ascending pathways are myelinated before 30 weeks of gestation, the descending pathways are immature, suggesting that preterm newborns (PTNBs) have a limited ability to modulate pain compared with full-term newborns and adults. In addition, prolonged exposure to painful events in the neonatal intensive care unit (NICU) may negatively affect the immature nervous system [1,2].

Acute pain induces adverse changes in the short term, whereas chronic pain alters the stress-response system and impacts neurodevelopment [3]. The immediate effects of painful procedures include increased heart rate, oxidative stress, cortisol levels, and reduced vagal activity. The long-term effects include diminished cortical thickness, reduced vagal activity, delayed perceptive-visual development, low IQ, internalization behavior [2], and changes in somatosensory and/or emotional components of pain response in adult life [4].

The concept of pain awareness in newborns led to the development of different instruments for assessing pain in the NICU; thus, allowing a better interpretation of pain to decide the needed analgesia [1,5,6]. The most important pain relief methods are non-pharmacological due to their safety, efficacy, and low cost. Oral sucrose (with and without non-nutritive suction) is the most used intervention for pain relief in newborns [6,7,8,9]. 

Among the non-pharmacological methods, gentle touch is effective in relieving pain. It consists of applying light and gentle pressure on the body of the newborn to stimulate low-threshold afferent fibers that influence the brain, autonomic nervous system, blood flow, and respiratory rate. The technique also provides immediate positive effects (e.g., comfort, decreased level of motor activity, and deep sleep), attenuates brain activity during painful procedures, increases oxygen saturation, and decreases heart rate and crying time [10,11].

Pain management is challenging in neonatal care [8]. Although sucrose is widely studied, it is still difficult to be implemented in health services in developing countries since it is produced in compounding pharmacies. In contrast, gentle touch is a low-cost and easy-to-apply method without side effects that can be performed by professionals and parents visiting the PTNBs in the NICU.

This study aimed to compare the effects of gentle touch and sucrose on pain relief during suctioning in PTNB.

## 2. Methods

This is a randomized crossover clinical trial in which PTNBs behaved as their own control. 

Data were collected in the NICU of a public hospital and maternity of Goiânia (Brazil) between March 2019 and June 2020. The study was approved by the research ethics committee involving human beings of Dona Iris Hospital and Maternity (CAAE: 2.894.555) on 14 September 2018 and registered in the Brazilian Clinical Trials Registry (RBR-75xk9k).

PTNBs of both sexes with low birth weight and admitted to the NICU of Dona Íris Hospital and Maternity of Goiânia participated in this study. Inclusion criteria comprised PTNBs (gestational age > 26 and <36 weeks and 5 days) with low birth weight (<2500 g); hemodynamically stable; minimal or no sedation; under mechanical ventilation, continuous positive airway pressure (CPAP), nasal cannula oxygen, or ambient air; under cardiac and respiratory monitoring; without respiratory discomfort or oxygen desaturation; and requiring suctioning during hospitalization in the NICU. Those with genetic syndromes, major malformations, and congenital infections were excluded.

Sample size was calculated (G*Power software version 3.1) based on randomized clinical trials that used pain reduction (assessed using pain assessment scales) as primary outcome [8,12]. Among the investigated studies, pain has reduced from 14.7% to 20.9% on the PIPP scale and 66.6% on the NIPS scale. The main objective of the study, and a pilot conducted with 10 PTNBs. The minimum sample size estimated was 45 PTNBs, considering an effect size of 0.5, power of 95%, and error of 5% (α = 0.05).

### 2.1. Randomization

PTNBs were submitted to three different suctioning procedures after the first suctioning without intervention (baseline). Right after, PTNBs were randomly allocated to receive the intervention with sucrose or gentle touch during suctioning 2 and 3. To ensure the quality of randomization, a list of random numbers was created in the Excel software (Microsoft2013, USA, 2013) to define the intervention sequence in each PTNB.

### 2.2. Blinding

The purpose of blinding was to keep the sucrose and gentle touch interventions unknown to researchers. The study aims were also not informed to evaluators who assessed pain using instruments when analyzing the videos. The statistician was blinded to avoid bias in the analysis and interpretation of results.

### 2.3. Variables 

The type of ventilation (spontaneous, nasal cannula oxygen, CPAP, non-invasive ventilation, or invasive mechanical ventilation), oxygen saturation, heart rate, duration of hospitalization, and date of suctioning were control variables. The behavior to painful stimuli and physiological parameters (heart rate, respiratory rate, and oxygen saturation) of the PTNB were dependent variables. Independent variables were gentle touch and 25% sucrose.

### 2.4. Interventions

Gentle touch was chosen for pain relief because it is a non-pharmacological method with relevant efficacy in PTNBs. It is easy-to-apply and consists of placing one hand over the head and the other hand over the abdomen of the PTNB, promoting a relaxing effect [12,13]. The pressure applied during the procedure was constant but did not restrict the body movement of the PTNB. Moreover, the hands of the professional were always in contact with the skin of the PTNB during the suctioning procedure.

The administration of 25% sucrose was chosen due to its efficacy in relieving acute pain in PTNBs [8,9,14,15,16]. The solution of 0.5 mL of 25% sucrose per kg was prepared according to Ribeiro et al. [17] and administered using a syringe without a needle.

### 2.5. Scales 

The Neonatal Infant Pain Scale (NIPS), adapted and translated to Brazilian Portuguese by Motta [18], was used to assess pain. This multidimensional and easy-to-apply scale was developed based on the Children’s Hospital of Eastern Ontario Pain Scale for newborns older than 24 weeks and with no neurological impairment. It assesses six indicators regarding behavioral response to acute pain (i.e., facial expression, cry, breathing pattern, motor activity of arms and legs, and state of arousal), and scores range between 0 and 7; a total score of >4 indicates pain [18,19,20]. 

The Premature Infant Pain Profile-Revised (PIPP-R) is a multidimensional scale that assesses acute pain in preterm and term newborns using seven behavioral indicators related to facial movements (brow bulge, eye squeeze, and nasolabial furrow) and physiological (heart rate and oxygen saturation) and contextual factors (gestational age and behavioral state). PIPP-R was translated and adapted to Brazilian Portuguese by Bueno et al. [21,22] and recently revised and validated to facilitate its use. Although indicators were maintained, the scoring method was modified. The score of each item ranges between 0 and 3, and the total pain score ranges between 0 and 21. Scores between 0 and 6 indicate mild pain; 7 to 12, moderate pain; and >12, severe pain [22]. 

### 2.6. Procedures

PTNBs with low birth weight and hospitalized in the NICU were identified after searches in medical records. Mothers or legal guardians were identified in the maternity and asked to participate; the interview was performed in the NICU. Due to the complexity of the study, most mothers or guardians asked for a joint reading of the informed consent form, which was signed after explaining all questions. The main characteristics of PTNBs were collected (e.g., birth type, birth weight, Apgar in the first and fifth minutes, and health complications during the neonatal phase). 

In the NICU where PTNBs were assessed, there are no pain assessment protocols or methods for pain relief during potentially painful procedures.

After signing the informed consent form, the physical therapist auscultated the PTNBs, identified the need for suctioning, and performed the suctioning procedure without intervention (baseline). None of the PTNBs was submitted to suctioning without clinical indication; thus, the procedure was only conducted on those routinely submitted to suctioning during hospitalization in the NICU. Intercurrences during suctioning (e.g., central cyanosis, abrupt oxygen desaturation, cardiorespiratory arrest, bradycardia, or apnea) led to the immediate interruption of data collection.

No intervention was conducted five minutes before the procedure. The mean duration of suctioning was 90 s, according to guidelines of the maternity, followed by a recovery of 30 s after the procedure.

PTNBs in the group starting with gentle touch received the intervention by a physiotherapist of the unit, whereas suctioning with sucrose was applied after 48 h (one minute before the suctioning procedure); the same criterion was applied for PTNBs who received sucrose first.

The list created in Microsoft Excel was consulted before the procedure to define the PTNBs receiving sucrose. For suctioning with sucrose, each PTNB received 0.5 mL of the solution in the anterior portion of the tongue one minute before the suctioning; after 48 h, they received the gentle touch intervention during suctioning. PTNBs remained in the incubator during the suctioning procedure with a rolled blanket around the body to promote a flexed posture.

After recordings, videos were edited and sent to evaluators to assess pain according to scales.

PTNBs were characterized according to the following data: general information, maternal information, conditions during pregnancy (pregnancy planning, prenatal, health complications, and exams), information about childbirth (type and clinical conditions of the birth), information about birth (gestational age, birth weight, and Apgar in the first and fifth minutes), neonatal complications, and clinical procedures conducted with the PTNB. After analyzing the medical history, the Clinical Risk Index for Babies II was applied to assess the clinical gravity of PTNBs. Information about hospital discharge was also collected [23].

Results from the pilot study indicated that the best angle for recording would be from above the incubator or heated crib, and arms and legs should be free during gentle touch to avoid bias in the assessment. The entire body was recorded, but focus was given to the face of the PTNB. 

The video was edited by an editor who did not participate in the research, and only the initials of the newborn, gestational age, and suctioning number (suctioning 1, suctioning 2, and suctioning 3) were included. Videos were sent through an online platform to two independent evaluators, who assessed each video separately and filled out the scores of the two scales. All information was further verified by the main researcher.

The study was conducted according to guidelines and regulatory standards of research involving human beings (resolution 466/12 of the National Health Council). According to the National Health Surveillance Agency (ANVISA-RDC36; 27 June 2012), phases I, II, III, and IV trials must be registered in the Brazilian Clinical Trials Registry database. This study was approved according to the register RBR-75xk9k.

### 2.7. Statistical Analysis

Data analysis was conducted using the Statistical Package for the Social Sciences software version 23.0 (SPSS, IBM Corp, Chicago, IL, USA) with a significance level of 5%. The statistical analysis was conducted in three stages.

Descriptive analysis and data normality: mean, standard deviation, median, and minimum and maximum values were calculated for continuous variables, whereas absolute and relative frequencies were provided for categorical variables. The Shapiro–Wilk test assessed data normality. Inferential analyses were based on non-parametric tests since outcomes were not normally distributed.

Reliability analysis: intraclass correlation coefficients (ICC) were calculated for individual and mean scores to assess the reliability of NIPS and PIPP-R between two researchers (A and B). ICC results were considered excellent (ICC > 0.90), very good (0.90 > ICC > 0.89), acceptable (0.79 > ICC > 0.71), or non-acceptable (ICC < 0.70) [24]. Cronbach’s alpha assessed the internal consistency between items of instruments. 

Analysis of groups with a paired sample: Friedman’s test compared NIPS and PIPP-R outcomes between suctioning procedures (suctioning without intervention, suctioning with gentle touch, and suctioning with sucrose). In the case of differences between interventions, a post hoc test for multiple comparisons (paired) was conducted, and the adjusted correction was applied according to the number of comparisons.

## 3. Results

**Figure 1:**
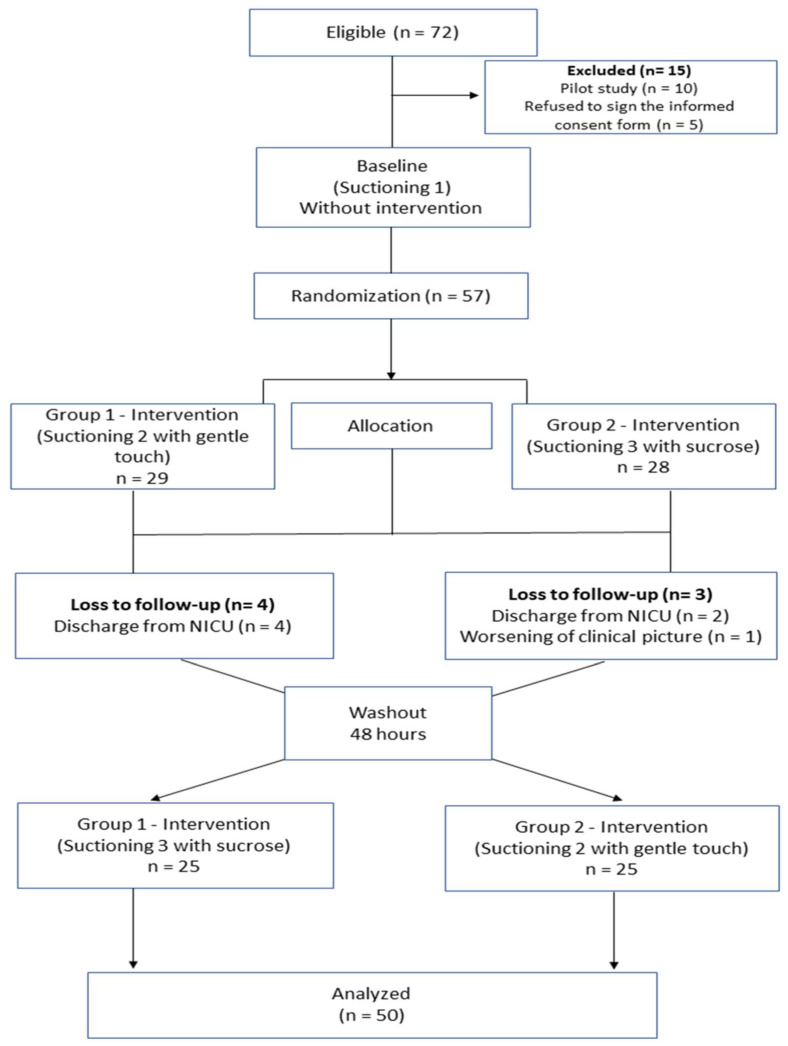
Study flowchart. Adapted from CONSORT 2010: extension to randomized crossover trials.

The sample comprised 50 PTNBs (Figure 1). Fifty PTNBs (48% males) with a mean gestational age of 28 weeks (24.42 to 35.14 weeks) and mean birth weight of 1050 g (595 to 2225 g) were enrolled in this study. Moreover, 58% of PTNBs were born from vaginal delivery and 42% from cesarean delivery. A total of 24% of PTNBs had complications during birth, and the median Apgar index in the first and fifth minute was 6 (0 to 9) and 8 (1 to 10), respectively. Mechanical ventilation was used by 82% of PTNBs, 74% used nasal CPAP, and 42% used oxygen (Table 1).

**Figure 2:**
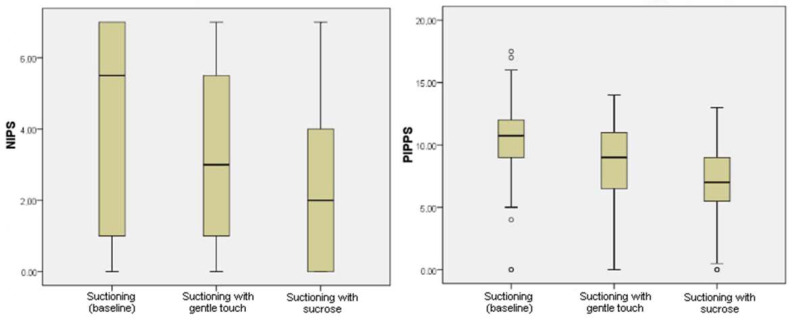
Total NIPS and PIPP-R scores during suctioning procedures (baseline, gentle touch, sucrose).

According to Figure 2, PTNBs felt pain during suctioning without intervention, represented by a NIPS total score of 5.0 and a PIPP-R score of 10.0 (moderate pain). NIPS and PIPP-R total scores were reduced when suctioning was conducted with gentle touch (3.0 and 10.0, respectively). The sucrose intervention also reduced NIPS and PIPP-R total scores to 2.5 and 8.5, respectively. Therefore, significant differences in the total score of the two pain scales were observed between the three suctioning procedures.

Table 2 shows a significant difference in the item cry of NIPS between the three suctioning procedures (*p* < 0.001). We also observed differences in the breathing pattern item (*p* < 0.001). 

Pain relief was also observed in the motor activity of arms, represented by a significant decrease (*p* < 0.005) induced by gentle touch and sucrose compared with suctioning without intervention. A significant difference was also observed in the motor activity of legs (*p* < 0.002), in which scores using gentle touch and sucrose were lower than without intervention. Regarding the state of arousal represented by a significant decrease (*p* < 0.001) in which scores using gentle touch and sucrose were lower than without intervention. 

The items brow bulge, eye squeeze, and nasolabial furrow of the PIPP-R were significantly lower (*p* < 0.001) in the suctioning with gentle touch and sucrose than without intervention (Table 2).

## 4. Discussion

This study assessed pain during suctioning procedures in PTNBs and the effects of gentle touch and sucrose on pain relief.

Mean (or median if you report medians) pain scores during the suction procedures were lowest in the sucrose condition, followed by gentle touch, and were highest in the no-treatment condition. Differences between treatment conditions were statistically significantly compared to no treatment (baseline).

PTNBs felt pain during suctioning, evidenced by the two pain assessment instruments (Table 2). In the study by Qiu et al., nasal suctioning was the second most painful procedure in PTNBs hospitalized in the NICU [13].

Only two studies assessed pain in PTNBs during suctioning. Alemdar and Tufekci [25] assessed pain in 62 PTNBs before, during, and after suctioning and observed pain in the control and intervention (maternal heart sounds) groups during and after the procedure. In the study by Fatollazade et al. [12], moderate to severe pain was observed during suctioning in 34 newborns. Our study observed a moderate pain during endotracheal and airway suctioning according to PIPP-R total score (Table 2).

We applied two non-pharmacological interventions to relieve pain during suctioning. The health professional provided comfort and safety during gentle touch by applying a soft and gentle pressure over the abdomen and head of PTNBs associated with postural alignment (medial line) [11,12,13,25]. Few studies reporting gentle touch for pain relief were found in the literature.

Gentle touch significantly reduced the state of arousal assessed using NIPS. However, no significant effect was observed in the behavioral state and physiological parameters of the PIPP-R scale compared to baseline (Table 2). This result corroborates a randomized clinical trial that assessed static and dynamic touch in 92 PTNBs for reducing physiological excitation (heart rate and oxygen saturation) and found no significant difference after static touch [26]. In the study by Fatollahzade et al. [12], gentle touch improved the physiological parameters and behavioral responses of PTNBs during suctioning in the first weeks of hospitalization in the NICU.

Gentle touch can attenuate cerebral activities during a painful procedure, increasing saturation and reducing heart rate and duration of crying [27]. Our study observed a significant difference in the cry item of NIPS between suctioning with gentle touch and without intervention. However, no significant differences in heart rate or oxygen saturation of the PIPP-R scale were observed among the conditions (Table 2).

The combined use of facilitated tucking and breast milk reduced pain in PTNBs (moderate and high to low pain) during heel puncture. Pain was reduced by 64% and 70.1%, respectively, compared with newborns who received routine care [28]. In our study, gentle touch and sucrose reduced pain, despite not reaching the minimum scores (Table 2). The association between interventions was not verified.

Sucrose relieved pain during suctioning, as demonstrated by significant differences between interventions and baseline. However, no significant difference was observed compared with gentle touch. In a randomized clinical trial conducted with 120 PTNBs, sucrose and the combination of sucrose and music reduced pain during and after 30 s of the heel puncture procedure compared with baseline [29]. 

Sucrose (25%; 0.5 mL/kg) was administered one minute before suctioning. The study by Stevens et al. [8] conducted with 250 preterm infants demonstrated that 0.1 mL of 24% sucrose was the minimal dose needed to relieve pain during the heel puncture procedure. However, this dose was not significantly different from 0.5 and 1.0 mL of sucrose in terms of effectiveness in reducing pain in preterm infants. Moreover, pain was not completely alleviated during the heel puncture procedure. 

A meta-analysis showed that doses between 0.005 and 0.5 mL of 25% sucrose significantly decreased PIPP-R scores. The most intense analgesic effects occurred by administering sucrose approximately two minutes before the painful stimulus, probably due to the release of endogenous opioids. Sucrose was effective for pain relief and presented minimal or no adverse effects [8]. 

A study conducted with 86 preterm infants assessed the effects of sucrose, non-nutritive sucking, and these two interventions combined on pain relief during heel puncture. The authors demonstrated the efficacy of sucrose for relieving pain; however, the relief was more intense when combining the interventions [30]. 

A previous study with 64 preterm infants assessed the effects of sucrose and kangaroo mother care on pain during heel puncture. Although sucrose decreased the PIPP-R score during and after the procedure, kangaroo mother care was more efficacious [31]. In our study, sucrose decreased NIPS and PIPP-R scores compared with baseline and gentle touch; however, no significant differences were observed between procedures. This corroborates with a meta-analysis conducted by Stevens et al. [8], who found no significant pain relief between sucrose and facilitated tucking.

The strengths of the study include the study design, randomization, blinding, and the specialized and experienced team of the NICU that accompanied all newborns during the study.

The limitations of this study included scratched incubators impairing the video recording. Furthermore, ventilatory support was not homogeneous within the sample since we assessed newborns at three different moments.

## 5. Conclusions

In conclusion, gentle touch and sucrose were efficacious for pain relief in PTNBs during suctioning, and no significant difference was observed between these interventions. Thus, gentle touch can be safely used in the NICU during painful procedures since it is a low-cost and easy-to-perform technique by professionals and family members. Our study provides evidence to improve care in the NICU and support health workers to assess pain and establish low-cost non-pharmacological methods to reduce pain in PTNBs submitted to painful procedures; therefore, reducing the deleterious effects in the short, medium, and long terms. Future studies are needed to assess the implementation of pain relief methods during painful procedures. 

## Figures and Tables

**Table 1:** Characteristics of the sample (n = 50).

Characteristics of Preterm Newborns	Values
Type of Birth f (%)	
Vaginal	29 (58)
Cesarean	21 (42)
Sex f (%)	
Female	26 (52)
Male	24 (48)
Birth weight (g)—Med (min–max)	1050 (886.3–1227.3)
Gestational age (weeks)—Med (min–max)	28 (26.7–30.3)
Clinical risk—Med (min–max)	
Apgar in the first minute (score)	6 (4–8)
Apgar in the fifth minute (score)	8 (7–9)
Cried at birth f (%)	36 (72)
Birth complications f (%)	12 (24)
Fetal distress f (%)	9 (18)
Neonatal health complications f (%)	
Infection f (%)	25 (50)
Apnea f (%)	8 (16)
Hyaline membrane disease f (%)	50 (100)
Bronchopulmonary dysplasia f (%)	1 (2)
Surfactant use f (%)	39 (78)
Antibiotic use f (%)	39 (78)
Phototherapy f (%)	25 (50)
Transfusion f (%)	10 (20)
Mechanical ventilation f (%)	41 (82)
Nasal CPAP f (%)	37 (74)
Oxygen support (%)	21 (42)
Hood f (%)	1 (2)
Transfontanellar ultrasound f (%)	
Altered	27 (54)
Normal	18 (36)
No information	5 (10)

Med = median; min = minimum value; max = maximal value; f = frequency; % = percentage; CPAP = continuous positive airway pressure.

**Table 2:** Median scores, standard deviation, confidence intervals, and comparison of NIPS and PIPP-R between the three conditions.

Indicator	Median (25–75% Interquartile Range)				Post Hoc		
	Baseline Suctioning	Suctioning with Gentle Touch	Suctioning with Sucrose	*p*-Value *	Baseline x Gentle Touch	Baseline x Sucrose	Gentle Touch x Sucrose
NIPS							
Facial expression	1.0 (0.5–1.0)	1.0 (0.5–1.0)	1.0 (0.5–1.0)	0.220			
Cry	0.5 (0.0–1.5)	0.5 (0.0–1.5)	0.0 (0.0–1.0)	<0.001 *	0.137	0.010 *	1
Breathing pattern	0.5 (0.5–1.0)	0.5 (0.0–1.0)	0.5 (0.0–1.0)	<0.001 *	0.581	0.007 *	0.24
Motor activity of arms	0.5 (0.0–1.0)	0.5 (0.5–1.0)	0.5 (0.0–0.8)	0.005 *	1	0.83	0.26
Motor activity of legs	0.5 (0.0–1.0)	0.5 (0.0–1.0)	0.5 (0.0–0.5)	0.002 *	0.154	0.032 *	1
State of arousal	0.5 (0.0–1.0)	0.5 (0.0–1.0)	0.0 (0.0–0.5)	<0.001 *	0.363	0.002 *	0.19
Total	5.0 (1.0–6.0)	3.0 (1.5–5.5)	2.5 (1.0–4.5)	<0.001 *	0.010 *	<0.001 *	0.24
PIPP-R							
Heart rate change	1.0 (0.3–1.8)	1.0 (1.0–2.5)	1.0 (0.0–2.0)	0.327			
Drop in oxygen saturation	1.0 (0.0–2.0)	1.0 (0.0–3.0)	0.5 (0.0–1.5)	0.157			
Brow bulge	1.0 (0.5–2.0)	1.0 (0.5–1.8)	1.0 (0.3–1.5)	<0.001 *	0.002 *	<0.001 *	0.48
Eye squeeze	1.0 (0.5–2.0)	1.0 (0.5–1.8)	1.0 (0.5–1.5)	<0.001 *	0.024 *	<0.001 *	0.32
Nasolabial furrow	1.0 (0.3–2.0)	0.5 (0.0–1.5)	0.5 (0.0–1.0)	<0.001 *	0.024 *	0.001 *	0.95
Gestational age	2.0 (2.0–3.0)	2.0 (2.0–3.0)	2.5 (2.0–3.0)	0.459			
Behavioral state	1.5 (1.0–2.5)	1.5 (1.0–2.0)	1.5 (1.0–2.5)	0.704			
Total	10.0 (7.5–12.0)	10.0 (8.5–12.0)	8.5 (7.0–10.5)	<0.001 *	0.121	<0.001 *	0.56

* Friedman’s test.

## Data Availability

Data will be made available upon request. If you are interested, you can ask the responsible researcher via email.
